# Supplementary material for: The pERKs of temporal order memory in mice
Source: Front Mol Neurosci. 2026 Aug 28;19:1912931. doi: 10.3389/fnmol.2026.1912931 (PMC13561817; doi:10.3389/fnmol.2026.1912931)
Supplement: Supplementary file 2 [file Data_Sheet_2.pdf]

Statistical Analysis

All statistical analyses were conducted using Generalized Linear Mixed Models (GLMMs) via the glmmTMB package in R. To accurately reflect the experimental design and avoid model overparameterization (singular fits), the inclusion of variables as fixed or random effects was carefully specified based on their structural characteristics.

For the behavioral models (TOM and TeNOR), home-cage identity and subject identity (when repeated measures were available) were included as nested random intercepts (1 | Cage / Mouse) to account for shared environmental and subject-level variance. Because sex represents a two-level variable mathematically unsuitable for estimating random variance components, it was explicitly modeled as a fixed effect. In the primary TOM analysis, sex was included as an interacting fixed effect to specifically test for sex-dependent differences in temporal memory. In contrast, for the TeNOR and molecular signaling models, where no sex-dependent interaction was hypothesized, sex was included as an additive fixed covariate (+ Sex) to control for potential baseline differences without over-complicating the interaction terms.

For post-hoc evaluations, Estimated Marginal Means (EMMs) were extracted using the emmeans package. To rigorously control the family-wise error rate across multiple tests, p-values for all-possible-pairwise comparisons between experimental groups (e.g., between TR1 and TR2) were adjusted using Tukey's method. Specific planned contrasts comparing experimental treatments directly against a single baseline (e.g., TR1 vs. HAB; TR2 vs. HAB) were adjusted using Dunnett's method. Finally, contrasts evaluating the Discrimination Index (DI) against the theoretical chance level (0.5) within each group were adjusted using the False Discovery Rate (FDR) procedure (Benjamini-Hochberg).

Correlation analyses linking behavioral exploration times to molecular expression profiles are explicitly defined as exploratory and hypothesis-generating; therefore, to preserve sensitivity to underlying biological signals, these specific correlational p-values are reported without adjustment for multiple comparisons.

Behavior (Figure 1)

1st TOM - DI

Model Structure: DI ~ Group * Sex + (1|Cage/Mouse)

Family: beta_family(link = 'logit')

DHARMa: Uniformity: p = 0.655 | Dispersion: p = 0.656 | Outliers: p = 1.000

Main Effects (ANOVA)

- Effect: Group | Statistic: χ^2 = 0.70, df = 2 | p-value: 0.7042 (ns)
- Effect: Sex | Statistic: χ^2 = 0.21, df = 1 | p-value: 0.6451 (ns)
- Effect: Group:Sex | Statistic: χ^2 = 0.42, df = 2 | p-value: 0.8094 (ns)

Test vs Random (0.5)

- Effect: 2m_ST - F
  - Magnitude: 11.3% [4.5 ; 17.6] | Statistic: z = 3.22 | p-value: 0.0038 (**)
- Effect: 3m_ST - F
  - Magnitude: 11.4% [4.6 ; 17.8] | Statistic: z = 3.26 | p-value: 0.0038 (**)
- Effect: 3m_LT - F
  - Magnitude: 7.8% [1.0 ; 14.4] | Statistic: z = 2.26 | p-value: 0.0241 (*)
- Effect: 2m_ST - M
  - Magnitude: 9.1% [2.6 ; 15.3] | Statistic: z = 2.73 | p-value: 0.0077 (**)
- Effect: 3m_ST - M
  - Magnitude: 10.2% [3.7 ; 16.3] | Statistic: z = 3.05 | p-value: 0.0046 (**)
- Effect: 3m_LT - M
  - Magnitude: 9.8% [3.3 ; 16.0] | Statistic: z = 2.94 | p-value: 0.0049 (**)

1st TOM - Total_exp_time

Model Structure: Total_exp_time ~ Group * Sex + (1|Cage/Mouse)

Family: gaussian

DHARMa: Uniformity: p = 0.291 | Dispersion: p = 0.376 | Outliers: p = 0.105

Main Effects (ANOVA)

- Effect: Group | Statistic: χ^2 = 30.36, df = 2 | p-value: < 0.0001 (***)
- Effect: Sex | Statistic: χ^2 = 0.71, df = 1 | p-value: 0.3991 (ns)
- Effect: Group:Sex | Statistic: χ^2 = 10.47, df = 2 | p-value: 0.0053 (**)

Post-Hoc Comparisons (Tukey)

- Effect: 2m_ST F - 3m_ST F

    - Magnitude: Diff(s) = -3.18 ± 3.73 | Statistic: z = -0.85 | p-value: 0.9573 (ns)
- Effect: 2m_ST F - 3m_LT F
    - Magnitude: Diff(s) = -19.19 ± 3.73 | Statistic: z = -5.14 | p-value: < 0.0001 (***)
- Effect: 2m_ST F - 2m_ST M
    - Magnitude: Diff(s) = 3.99 ± 4.73 | Statistic: z = 0.84 | p-value: 0.9594 (ns)
- Effect: 2m_ST F - 3m_ST M
    - Magnitude: Diff(s) = 1.36 ± 4.73 | Statistic: z = 0.29 | p-value: 0.9997 (ns)
- Effect: 2m_ST F - 3m_LT M
    - Magnitude: Diff(s) = -0.45 ± 4.73 | Statistic: z = -0.09 | p-value: 1.000 (ns)
- Effect: 3m_ST F - 3m_LT F
    - Magnitude: Diff(s) = -16.00 ± 3.73 | Statistic: z = -4.29 | p-value: 0.0003 (***)
- Effect: 3m_ST F - 2m_ST M
    - Magnitude: Diff(s) = 7.17 ± 4.73 | Statistic: z = 1.52 | p-value: 0.6539 (ns)
- Effect: 3m_ST F - 3m_ST M
    - Magnitude: Diff(s) = 4.54 ± 4.73 | Statistic: z = 0.96 | p-value: 0.9305 (ns)
- Effect: 3m_ST F - 3m_LT M
    - Magnitude: Diff(s) = 2.74 ± 4.73 | Statistic: z = 0.58 | p-value: 0.9925 (ns)
- Effect: 3m_LT F - 2m_ST M
    - Magnitude: Diff(s) = 23.18 ± 4.73 | Statistic: z = 4.90 | p-value: < 0.0001 (***)
- Effect: 3m_LT F - 3m_ST M
    - Magnitude: Diff(s) = 20.54 ± 4.73 | Statistic: z = 4.34 | p-value: 0.0002 (***)
- Effect: 3m_LT F - 3m_LT M
    - Magnitude: Diff(s) = 18.74 ± 4.73 | Statistic: z = 3.96 | p-value: 0.0011 (**)
- Effect: 2m_ST M - 3m_ST M
    - Magnitude: Diff(s) = -2.63 ± 3.57 | Statistic: z = -0.74 | p-value: 0.9774 (ns)
- Effect: 2m_ST M - 3m_LT M
    - Magnitude: Diff(s) = -4.44 ± 3.57 | Statistic: z = -1.24 | p-value: 0.8162 (ns)
- Effect: 3m_ST M - 3m_LT M
    - Magnitude: Diff(s) = -1.81 ± 3.57 | Statistic: z = -0.51 | p-value: 0.9960 (ns)

Behavior (Figure 2)

2nd TOM - DI

Model Structure: DI ~ Sex + (1|Cage)

Family: beta_family(link = 'logit')

DHARMa: Uniformity: p = 0.951 | Dispersion: p = 0.744 | Outliers: p = 1.000

- ANOVA:
    - Effect: Sex | Statistic: χ^2 = 0.18, df = 1 | p-value: 0.6703 (ns)
- Test vs Random: (0.5)
    - Effect: F
    - Magnitude: 13.6% [6.8 ; 20.0]
    - Statistic: z = 3.83

    - p-value: 0.0003 (Significance: ***)
- Test vs Random: (0.5)
    - Effect: M
    - Magnitude: 11.4% [2.9 ; 19.2]
    - Statistic: $z = 2.62$
    - p-value: 0.0089 (Significance: **)

2nd TOM - Total_exp_time

Model Structure: Total_exp_time ~ Sex + (1|Cage)

Family: gaussian

DHARMa: Uniformity: $p = 0.595$ | Dispersion: $p = 0.592$ | Outliers: $p = 1.000$

- ANOVA:
    - Effect: Sex
    - Statistic: $\chi^2 = 0.0$, df = 1
    - p-value: 0.9589 (Significance: ns)
- Post-Hoc (Tukey):
    - Effect: F - M
    - Magnitude: Diff(s) = -0.20 ± 3.78
    - Statistic: $z = -0.05$
    - p-value: 0.9589 (Significance: ns)

Western blot analysis

PFC cytosolic phospho ERK (Figure 3C)

Model Structure: Ratio ~ Training * Wait + Sex + (1|Cage)

Family: Gamma(link = 'log')

DHARMa: Uniformity: p = 0.641 | Dispersion: p = 0.792 | Outliers: p = 1.000

Main Effects (ANOVA)

- Effect: Sex | Statistic: $\chi^2 = 0.0$, df = 1 | p-value: 0.9647 (ns)
- Effect: Training | Statistic: $\chi^2 = 0.72$, df = 2 | p-value: 0.6979 (ns)
- Effect: Wait | Statistic: $\chi^2 = 5.58$, df = 2 | p-value: 0.0613 (.)
- Effect: Training:Wait | Statistic: $\chi^2 = 15.15$, df = 2 | p-value: 0.0005 (***)

Post-Hoc vs Global Baseline (0h_HAB)

- Effect: 0h | TR1 | vs 0h_HAB
  - Magnitude: -0.054 [-0.328 ; 0.22] | Statistic: z = -0.51 | p-value: 0.9638 (ns)
- Effect: 1h | TR1 | vs 0h_HAB
  - Magnitude: 0.393 [0.048 ; 0.738] | Statistic: z = 2.94 | p-value: 0.0178 (*)
- Effect: 1h | TR2 | vs 0h_HAB
  - Magnitude: 0.039 [-0.249 ; 0.327] | Statistic: z = 0.35 | p-value: 0.9879 (ns)
- Effect: 2h | HAB | vs 0h_HAB
  - Magnitude: 0.040 [-0.266 ; 0.345] | Statistic: z = 0.33 | p-value: 0.9893 (ns)
- Effect: 2h | TR1 | vs 0h_HAB
  - Magnitude: 0.072 [-0.24 ; 0.384] | Statistic: z = 0.60 | p-value: 0.9434 (ns)
- Effect: 2h | TR2 | vs 0h_HAB
  - Magnitude: 0.441 [0.058 ; 0.823] | Statistic: z = 2.97 | p-value: 0.0160 (*)

Post-Hoc Intra-Wait (Pairwise)

- Effect: 0h | HAB - TR1
  - Magnitude: 0.054 [-0.154 ; 0.262] | Statistic: z = 0.51 | p-value: 0.6117 (ns)
- Effect: 1h | TR1 - TR2
  - Magnitude: 0.354 [0.088 ; 0.620] | Statistic: z = 2.61 | p-value: 0.0090 (**)
- Effect: 2h | HAB - TR1
  - Magnitude: -0.033 [-0.336 ; 0.271] | Statistic: z = -0.25 | p-value: 0.8021 (ns)
- Effect: 2h | HAB - TR2
  - Magnitude: -0.401 [-0.765 ; -0.037] | Statistic: z = -2.58 | p-value: 0.0282 (*)
- Effect: 2h | TR1 - TR2
  - Magnitude: -0.369 [-0.737 ; -0.001] | Statistic: z = -2.35 | p-value: 0.0282 (*)

PFC cytosolic total ERK (Figure S3)

Model Structure: Ratio ~ Training * Wait + Sex + (1|Cage)

Family: Gamma(link = 'log')

DHARMa: Uniformity: p = 0.562 | Dispersion: p = 0.840 | Outliers: p = 1.000

Main Effects (ANOVA)

- Effect: Sex | Statistic: χ^2 = 0.58, df = 1 | p-value: 0.4450 (ns)
- Effect: Training | Statistic: χ^2 = 2.37, df = 2 | p-value: 0.3056 (ns)
- Effect: Wait | Statistic: χ^2 = 0.64, df = 2 | p-value: 0.7262 (ns)
- Effect: Training:Wait | Statistic: χ^2 = 1.21, df = 2 | p-value: 0.5463 (ns)

Post-Hoc vs Global Baseline (0h_HAB)

- Effect: 0h | TR1 | vs 0h_HAB
  - Magnitude: -0.098 [-0.364 ; 0.168] | Statistic: z = -0.95 | p-value: 0.8012 (ns)
- Effect: 1h | TR1 | vs 0h_HAB
  - Magnitude: -0.072 [-0.342 ; 0.198] | Statistic: z = -0.69 | p-value: 0.9150 (ns)
- Effect: 1h | TR2 | vs 0h_HAB
  - Magnitude: 0.089 [-0.205 ; 0.383] | Statistic: z = 0.78 | p-value: 0.8801 (ns)
- Effect: 2h | HAB | vs 0h_HAB
  - Magnitude: 0.052 [-0.254 ; 0.358] | Statistic: z = 0.44 | p-value: 0.9763 (ns)
- Effect: 2h | TR1 | vs 0h_HAB
  - Magnitude: -0.008 [-0.304 ; 0.288] | Statistic: z = -0.07 | p-value: 0.9999 (ns)
- Effect: 2h | TR2 | vs 0h_HAB
  - Magnitude: -0.027 [-0.319 ; 0.266] | Statistic: z = -0.24 | p-value: 0.9961 (ns)

Post-Hoc Intra-Wait (Pairwise)

- Effect: 0h | HAB - TR1
  - Magnitude: 0.098 [-0.104 ; 0.300] | Statistic: z = 0.95 | p-value: 0.3312 (ns)
- Effect: 1h | TR1 - TR2
  - Magnitude: -0.161 [-0.377 ; 0.055] | Statistic: z = -1.46 | p-value: 0.1412 (ns)
- Effect: 2h | HAB - TR1
  - Magnitude: 0.060 [-0.231 ; 0.351] | Statistic: z = 0.48 | p-value: 0.8846 (ns)
- Effect: 2h | HAB - TR2
  - Magnitude: 0.079 [-0.210 ; 0.367] | Statistic: z = 0.64 | p-value: 0.8846 (ns)
- Effect: 2h | TR1 - TR2
  - Magnitude: 0.019 [-0.261 ; 0.298] | Statistic: z = 0.16 | p-value: 0.8846 (ns)

PFC cytosolic MKP3 (Figure 3D)

Model Structure: Ratio ~ Training * Wait + Sex + (1|Cage)

Family: Gamma(link = 'log')

DHARMa: Uniformity: p = 0.326 | Dispersion: p = 0.640 | Outliers: p = 0.401

Main Effects (ANOVA)

- Effect: Sex | Statistic: χ^2 = 0.78, df = 1 | p-value: 0.3769 (ns)
- Effect: Training | Statistic: χ^2 = 1.43, df = 2 | p-value: 0.4889 (ns)
- Effect: Wait | Statistic: χ^2 = 5.88, df = 2 | p-value: 0.0530 (.)
- Effect: Training:Wait | Statistic: χ^2 = 7.0, df = 2 | p-value: 0.0302 (*)

Post-Hoc vs Global Baseline (0h_HAB)

- Effect: 0h | TR1 | vs 0h_HAB
    - Magnitude: 0.020 [-0.251 ; 0.291] | Statistic: z = 0.19 | p-value: 0.9980 (ns)
- Effect: 1h | TR1 | vs 0h_HAB
    - Magnitude: 0.058 [-0.219 ; 0.335] | Statistic: z = 0.54 | p-value: 0.9566 (ns)
- Effect: 1h | TR2 | vs 0h_HAB
    - Magnitude: 0.381 [0.053 ; 0.709] | Statistic: z = 3.00 | p-value: 0.0146 (*)
- Effect: 2h | HAB | vs 0h_HAB
    - Magnitude: 0.095 [-0.206 ; 0.396] | Statistic: z = 0.82 | p-value: 0.8652 (ns)
- Effect: 2h | TR1 | vs 0h_HAB
    - Magnitude: 0.047 [-0.246 ; 0.339] | Statistic: z = 0.41 | p-value: 0.9804 (ns)
- Effect: 2h | TR2 | vs 0h_HAB
    - Magnitude: -0.060 [-0.334 ; 0.215] | Statistic: z = -0.56 | p-value: 0.9517 (ns)

Post-Hoc Intra-Wait (Pairwise)

- Effect: 0h | HAB - TR1
    - Magnitude: -0.020 [-0.226 ; 0.186] | Statistic: z = -0.19 | p-value: 0.8860 (ns)
- Effect: 1h | TR1 - TR2
    - Magnitude: -0.323 [-0.577 ; -0.069] | Statistic: z = -2.5 | p-value: 0.0123 (*)
- Effect: 2h | HAB - TR1
    - Magnitude: 0.049 [-0.244 ; 0.342] | Statistic: z = 0.39 | p-value: 0.6769 (ns)
- Effect: 2h | HAB - TR2
    - Magnitude: 0.155 [-0.123 ; 0.433] | Statistic: z = 1.31 | p-value: 0.5212 (ns)
- Effect: 2h | TR1 - TR2
    - Magnitude: 0.106 [-0.164 ; 0.377] | Statistic: z = 0.92 | p-value: 0.5212 (ns)

PFC nuclear phospho ERK (Figure S2)

Model Structure: Ratio ~ Training * Wait + Sex + (1|Cage)

Family: Gamma(link = 'log')

DHARMa: Uniformity: p = 0.859 | Dispersion: p = 0.848 | Outliers: p = 0.401

Main Effects (ANOVA)

- Effect: Sex | Statistic: $\chi^2 = 0.26$, df = 1 | p-value: 0.6123 (ns)
- Effect: Training | Statistic: $\chi^2 = 5.82$, df = 2 | p-value: 0.0545 (.)
- Effect: Wait | Statistic: $\chi^2 = 1.86$, df = 2 | p-value: 0.3937 (ns)
- Effect: Training:Wait | Statistic: $\chi^2 = 9.43$, df = 2 | p-value: 0.0089 (**)

Post-Hoc vs Global Baseline (0h_HAB)

- Effect: 0h | TR1 | vs 0h_HAB
  - Magnitude: 0.163 [-0.148 ; 0.474] | Statistic: z = 1.35 | p-value: 0.5554 (ns)
- Effect: 1h | TR1 | vs 0h_HAB
  - Magnitude: 0.498 [0.132 ; 0.864] | Statistic: z = 3.51 | p-value: 0.0025 (**)
- Effect: 1h | TR2 | vs 0h_HAB
  - Magnitude: 0.052 [-0.242 ; 0.346] | Statistic: z = 0.46 | p-value: 0.9733 (ns)
- Effect: 2h | HAB | vs 0h_HAB
  - Magnitude: 0.017 [-0.29 ; 0.324] | Statistic: z = 0.14 | p-value: 0.9991 (ns)
- Effect: 2h | TR1 | vs 0h_HAB
  - Magnitude: 0.081 [-0.237 ; 0.398] | Statistic: z = 0.66 | p-value: 0.9257 (ns)
- Effect: 2h | TR2 | vs 0h_HAB
  - Magnitude: 0.261 [-0.09 ; 0.611] | Statistic: z = 1.92 | p-value: 0.2313 (ns)

Post-Hoc Intra-Wait (Pairwise)

- Effect: 0h | HAB - TR1
  - Magnitude: -0.163 [-0.399 ; 0.073] | Statistic: z = -1.35 | p-value: 0.1765 (ns)
- Effect: 1h | TR1 - TR2
  - Magnitude: 0.446 [0.163 ; 0.729] | Statistic: z = 3.09 | p-value: 0.0019 (**)
- Effect: 2h | HAB - TR1
  - Magnitude: -0.064 [-0.369 ; 0.242] | Statistic: z = -0.49 | p-value: 0.6180 (ns)
- Effect: 2h | HAB - TR2
  - Magnitude: -0.244 [-0.577 ; 0.09] | Statistic: z = -1.71 | p-value: 0.2550 (ns)
- Effect: 2h | TR1 - TR2
  - Magnitude: -0.18 [-0.522 ; 0.162] | Statistic: z = -1.23 | p-value: 0.3258 (ns)

PFC nuclear total ERK (Figure S2)

Model Structure: Ratio ~ Training * Wait + Sex + (1|Cage)

Family: Gamma(link = 'log')

DHARMa: Uniformity: p = 0.998 | Dispersion: p = 0.848 | Outliers: p = 1.000

Main Effects (ANOVA)

- Effect: Sex | Statistic: χ^2 = 0.61, df = 1 | p-value: 0.4353 (ns)
- Effect: Training | Statistic: χ^2 = 2.09, df = 2 | p-value: 0.3520 (ns)
- Effect: Wait | Statistic: χ^2 = 3.96, df = 2 | p-value: 0.1381 (ns)
- Effect: Training:Wait | Statistic: χ^2 = 0.46, df = 2 | p-value: 0.7955 (ns)

Post-Hoc vs Global Baseline (0h_HAB)

- Effect: 0h | TR1 | vs 0h_HAB
  - Magnitude: 0.093 [-0.136 ; 0.322] | Statistic: z = 1.05 | p-value: 0.7459 (ns)
- Effect: 1h | TR1 | vs 0h_HAB
  - Magnitude: 0.169 [-0.069 ; 0.407] | Statistic: z = 1.84 | p-value: 0.2698 (ns)
- Effect: 1h | TR2 | vs 0h_HAB
  - Magnitude: 0.09 [-0.138 ; 0.318] | Statistic: z = 1.02 | p-value: 0.7644 (ns)
- Effect: 2h | HAB | vs 0h_HAB
  - Magnitude: 0.019 [-0.215 ; 0.253] | Statistic: z = 0.21 | p-value: 0.9972 (ns)
- Effect: 2h | TR1 | vs 0h_HAB
  - Magnitude: 0.028 [-0.207 ; 0.263] | Statistic: z = 0.31 | p-value: 0.9916 (ns)
- Effect: 2h | TR2 | vs 0h_HAB
  - Magnitude: -0.045 [-0.27 ; 0.181] | Statistic: z = -0.51 | p-value: 0.9635 (ns)

Post-Hoc Intra-Wait (Pairwise)

- Effect: 0h | HAB - TR1
  - Magnitude: -0.093 [-0.267 ; 0.081] | Statistic: z = -1.05 | p-value: 0.2855 (ns)
- Effect: 1h | TR1 - TR2
  - Magnitude: 0.079 [-0.109 ; 0.267] | Statistic: z = 0.83 | p-value: 0.4090 (ns)
- Effect: 2h | HAB - TR1
  - Magnitude: -0.009 [-0.235 ; 0.218] | Statistic: z = -0.09 | p-value: 0.9238 (ns)
- Effect: 2h | HAB - TR2
  - Magnitude: 0.064 [-0.155 ; 0.282] | Statistic: z = 0.68 | p-value: 0.7453 (ns)
- Effect: 2h | TR1 - TR2
  - Magnitude: 0.073 [-0.147 ; 0.292] | Statistic: z = 0.77 | p-value: 0.7453 (ns)

HIP cytosolic phospho ERK (Figure S3)

Model Structure: Ratio ~ Training * Wait + Sex + (1|Cage)

Family: Gamma(link = 'log')

DHARMa: Uniformity: p = 0.637 | Dispersion: p = 0.808 | Outliers: p = 1.000

Main Effects (ANOVA)

- Effect: Sex | Statistic: χ^2 = 0.2, df = 1 | p-value: 0.6517 (ns)
- Effect: Training | Statistic: χ^2 = 5.21, df = 2 | p-value: 0.0740 (.)
- Effect: Wait | Statistic: χ^2 = 2.15, df = 2 | p-value: 0.3408 (ns)
- Effect: Training:Wait | Statistic: χ^2 = 0.33, df = 2 | p-value: 0.8459 (ns)

Post-Hoc vs Global Baseline (0h_HAB)

- Effect: 0h | TR1 | vs 0h_HAB
    - Magnitude: 0.046 [-0.201 ; 0.293] | Statistic: z = 0.48 | p-value: 0.9691 (ns)
- Effect: 1h | TR1 | vs 0h_HAB
    - Magnitude: -0.040 [-0.277 ; 0.197] | Statistic: z = -0.44 | p-value: 0.9767 (ns)
- Effect: 1h | TR2 | vs 0h_HAB
    - Magnitude: 0.068 [-0.182 ; 0.318] | Statistic: z = 0.7 | p-value: 0.9103 (ns)
- Effect: 2h | HAB | vs 0h_HAB
    - Magnitude: -0.065 [-0.321 ; 0.191] | Statistic: z = -0.66 | p-value: 0.9256 (ns)
- Effect: 2h | TR1 | vs 0h_HAB
    - Magnitude: 0.062 [-0.214 ; 0.338] | Statistic: z = 0.58 | p-value: 0.9473 (ns)
- Effect: 2h | TR2 | vs 0h_HAB
    - Magnitude: 0.171 [-0.123 ; 0.464] | Statistic: z = 1.5 | p-value: 0.4608 (ns)

Post-Hoc Intra-Wait (Pairwise)

- Effect: 0h | HAB - TR1
    - Magnitude: -0.046 [-0.234 ; 0.142] | Statistic: z = -0.48 | p-value: 0.6337 (ns)
- Effect: 1h | TR1 - TR2
    - Magnitude: -0.108 [-0.294 ; 0.078] | Statistic: z = -1.14 | p-value: 0.2655 (ns)
- Effect: 2h | HAB - TR1
    - Magnitude: -0.127 [-0.389 ; 0.135] | Statistic: z = -1.14 | p-value: 0.3812 (ns)
- Effect: 2h | HAB - TR2
    - Magnitude: -0.236 [-0.513 ; 0.042] | Statistic: z = -1.99 | p-value: 0.1393 (ns)
- Effect: 2h | TR1 - TR2
    - Magnitude: -0.109 [-0.401 ; 0.184] | Statistic: z = -0.87 | p-value: 0.3812 (ns)

HIP cytosolic total ERK (Figure S3)

Model Structure: Ratio ~ Training * Wait + Sex + (1|Cage)

Family: Gamma(link = 'log')

DHARMa: Uniformity: p = 0.905 | Dispersion: p = 0.912 | Outliers: p = 0.386

Main Effects (ANOVA)

- Effect: Sex | Statistic: χ^2 = 1.81, df = 1 | p-value: 0.1787 (ns)
- Effect: Training | Statistic: χ^2 = 5.95, df = 2 | p-value: 0.0510 (.)
- Effect: Wait | Statistic: χ^2 = 1.91, df = 2 | p-value: 0.3849 (ns)
- Effect: Training:Wait | Statistic: χ^2 = 0.08, df = 2 | p-value: 0.9629 (ns)

Post-Hoc vs Global Baseline (0h_HAB)

- Effect: 0h | TR1 | vs 0h_HAB
    - Magnitude: -0.151 [-0.381 ; 0.079] | Statistic: z = -1.69 | p-value: 0.3463 (ns)
- Effect: 1h | TR1 | vs 0h_HAB
    - Magnitude: -0.112 [-0.347 ; 0.123] | Statistic: z = -1.23 | p-value: 0.6346 (ns)
- Effect: 1h | TR2 | vs 0h_HAB
    - Magnitude: -0.042 [-0.285 ; 0.201] | Statistic: z = -0.45 | p-value: 0.9750 (ns)
- Effect: 2h | HAB | vs 0h_HAB
    - Magnitude: -0.031 [-0.299 ; 0.238] | Statistic: z = -0.29 | p-value: 0.9926 (ns)
- Effect: 2h | TR1 | vs 0h_HAB
    - Magnitude: -0.181 [-0.427 ; 0.066] | Statistic: z = -1.89 | p-value: 0.2430 (ns)
- Effect: 2h | TR2 | vs 0h_HAB
    - Magnitude: -0.146 [-0.397 ; 0.105] | Statistic: z = -1.5 | p-value: 0.4586 (ns)

Post-Hoc Intra-Wait (Pairwise)

- Effect: 0h | HAB - TR1
    - Magnitude: 0.151 [-0.024 ; 0.326] | Statistic: z = 1.74 | p-value: 0.0816 (.)
- Effect: 1h | TR1 - TR2
    - Magnitude: -0.07 [-0.244 ; 0.104] | Statistic: z = -0.82 | p-value: 0.4145 (ns)
- Effect: 2h | HAB - TR1
    - Magnitude: 0.15 [-0.092 ; 0.392] | Statistic: z = 1.44 | p-value: 0.4024 (ns)
- Effect: 2h | HAB - TR2
    - Magnitude: 0.116 [-0.131 ; 0.362] | Statistic: z = 1.11 | p-value: 0.4024 (ns)
- Effect: 2h | TR1 - TR2
    - Magnitude: -0.034 [-0.261 ; 0.192] | Statistic: z = -0.34 | p-value: 0.7373 (ns)

HIP cytosolic MKP3 (Figure S3)

Model Structure: Ratio ~ Training * Wait + Sex + (1|Cage)

Family: Gamma(link = 'log')

DHARMa: Uniformity: p = 0.744 | Dispersion: p = 0.800 | Outliers: p = 1.000

Main Effects (ANOVA)

- Effect: Sex | Statistic: χ^2 = 0.82, df = 1 | p-value: 0.5925 (ns)
- Effect: Training | Statistic: χ^2 = 3.09, df = 2 | p-value: 0.2131 (ns)
- Effect: Wait | Statistic: χ^2 = 0.17, df = 2 | p-value: 0.9169 (ns)
- Effect: Training:Wait | Statistic: χ^2 = 1.05, df = 2 | p-value: 0.5925 (ns)

Post-Hoc vs Global Baseline (0h_HAB)

- Effect: 0h | TR1 | vs 0h_HAB
  - Magnitude: -0.23 [-0.662 ; 0.202] | Statistic: z = -1.38 | p-value: 0.5408 (ns)
- Effect: 1h | TR1 | vs 0h_HAB
  - Magnitude: -0.208 [-0.644 ; 0.228] | Statistic: z = -1.23 | p-value: 0.6342 (ns)
- Effect: 1h | TR2 | vs 0h_HAB
  - Magnitude: 0.032 [-0.455 ; 0.519] | Statistic: z = 0.17 | p-value: 0.9986 (ns)
- Effect: 2h | HAB | vs 0h_HAB
  - Magnitude: -0.049 [-0.564 ; 0.465] | Statistic: z = -0.25 | p-value: 0.9955 (ns)
- Effect: 2h | TR1 | vs 0h_HAB
  - Magnitude: -0.104 [-0.603 ; 0.396] | Statistic: z = -0.54 | p-value: 0.9578 (ns)
- Effect: 2h | TR2 | vs 0h_HAB
  - Magnitude: -0.107 [-0.605 ; 0.392] | Statistic: z = -0.55 | p-value: 0.9542 (ns)

Post-Hoc Intra-Wait (Pairwise)

- Effect: 0h | HAB - TR1
  - Magnitude: 0.23 [-0.098 ; 0.558] | Statistic: z = 1.38 | p-value: 0.1476 (ns)
- Effect: 1h | TR1 - TR2
  - Magnitude: -0.24 [-0.577 ; 0.097] | Statistic: z = -1.4 | p-value: 0.1771 (ns)
- Effect: 2h | HAB - TR1
  - Magnitude: 0.054 [-0.43 ; 0.538] | Statistic: z = 0.26 | p-value: 0.9814 (ns)
- Effect: 2h | HAB - TR2
  - Magnitude: 0.057 [-0.426 ; 0.54] | Statistic: z = 0.28 | p-value: 0.9814 (ns)
- Effect: 2h | TR1 - TR2
  - Magnitude: 0.003 [-0.467 ; 0.473] | Statistic: z = 0.01 | p-value: 0.9814 (ns)

HIP nuclear phospho ERK (Figure S2)

Model Structure: Ratio ~ Training * Wait + Sex + (1|Cage)

Family: Gamma(link = 'log')

DHARMa: Uniformity: p = 0.226 | Dispersion: p = 0.496 | Outliers: p = 1.000

Main Effects (ANOVA)

- Effect: Sex | Statistic: χ^2 = 0.09, df = 1 | p-value: 0.7616 (ns)
- Effect: Training | Statistic: χ^2 = 0.08, df = 2 | p-value: 0.9606 (ns)
- Effect: Wait | Statistic: χ^2 = 1.88, df = 2 | p-value: 0.3898 (ns)
- Effect: Training:Wait | Statistic: χ^2 = 2.54, df = 2 | p-value: 0.2802 (ns)

Post-Hoc vs Global Baseline (0h_HAB)

- Effect: 0h | TR1 | vs 0h_HAB
  - Magnitude: -0.067 [-0.311 ; 0.177] | Statistic: z = -0.71 | p-value: 0.9083 (ns)
- Effect: 1h | TR1 | vs 0h_HAB
  - Magnitude: -0.001 [-0.253 ; 0.251] | Statistic: z = -0.01 | p-value: 1.0000 (ns)
- Effect: 1h | TR2 | vs 0h_HAB
  - Magnitude: -0.077 [-0.32 ; 0.166] | Statistic: z = -0.82 | p-value: 0.8649 (ns)
- Effect: 2h | HAB | vs 0h_HAB
  - Magnitude: -0.007 [-0.274 ; 0.259] | Statistic: z = -0.07 | p-value: 0.9999 (ns)
- Effect: 2h | TR1 | vs 0h_HAB
  - Magnitude: 0.017 [-0.253 ; 0.288] | Statistic: z = 0.17 | p-value: 0.9986 (ns)
- Effect: 2h | TR2 | vs 0h_HAB
  - Magnitude: 0.124 [-0.163 ; 0.41] | Statistic: z = 1.12 | p-value: 0.7072 (ns)

Post-Hoc Intra-Wait (Pairwise)

- Effect: 0h | HAB - TR1
  - Magnitude: 0.067 [-0.118 ; 0.252] | Statistic: z = 0.71 | p-value: 0.4761 (ns)
- Effect: 1h | TR1 - TR2
  - Magnitude: 0.076 [-0.108 ; 0.26] | Statistic: z = 0.81 | p-value: 0.4158 (ns)
- Effect: 2h | HAB - TR1
  - Magnitude: -0.025 [-0.283 ; 0.233] | Statistic: z = -0.23 | p-value: 0.8177 (ns)
- Effect: 2h | HAB - TR2
  - Magnitude: -0.131 [-0.403 ; 0.14] | Statistic: z = -1.13 | p-value: 0.5461 (ns)
- Effect: 2h | TR1 - TR2
  - Magnitude: -0.106 [-0.381 ; 0.168] | Statistic: z = -0.91 | p-value: 0.5461 (ns)

HIP nuclear total ERK (Figure S2)

Model Structure: Ratio ~ Training * Wait + Sex + (1|Cage)

Family: Gamma(link = 'log')

DHARMa: Uniformity: p = 0.279 | Dispersion: p = 0.768 | Outliers: p = 1.000

Main Effects (ANOVA)

- Effect: Sex | Statistic: χ^2 = 0.02, df = 1 | p-value: 0.8829 (ns)
- Effect: Training | Statistic: χ^2 = 0.66, df = 2 | p-value: 0.7198 (ns)
- Effect: Wait | Statistic: χ^2 = 1.93, df = 2 | p-value: 0.3811 (ns)
- Effect: Training:Wait | Statistic: χ^2 = 0.54, df = 2 | p-value: 0.7618 (ns)

Post-Hoc vs Global Baseline (0h_HAB)

- Effect: 0h | TR1 | vs 0h_HAB
  - Magnitude: 0.094 [-0.243 ; 0.431] | Statistic: z = 0.72 | p-value: 0.9043 (ns)
- Effect: 1h | TR1 | vs 0h_HAB
  - Magnitude: 0.155 [-0.193 ; 0.503] | Statistic: z = 1.15 | p-value: 0.6865 (ns)
- Effect: 1h | TR2 | vs 0h_HAB
  - Magnitude: 0.106 [-0.233 ; 0.445] | Statistic: z = 0.81 | p-value: 0.8701 (ns)
- Effect: 2h | HAB | vs 0h_HAB
  - Magnitude: 0.048 [-0.302 ; 0.399] | Statistic: z = 0.36 | p-value: 0.9871 (ns)
- Effect: 2h | TR1 | vs 0h_HAB
  - Magnitude: 0.014 [-0.329 ; 0.358] | Statistic: z = 0.11 | p-value: 0.9996 (ns)
- Effect: 2h | TR2 | vs 0h_HAB
  - Magnitude: -0.053 [-0.383 ; 0.277] | Statistic: z = -0.41 | p-value: 0.9799 (ns)

Post-Hoc Intra-Wait (Pairwise)

- Effect: 0h | HAB - TR1
  - Magnitude: -0.094 [-0.35 ; 0.162] | Statistic: z = -0.72 | p-value: 0.4691 (ns)
- Effect: 1h | TR1 - TR2
  - Magnitude: 0.049 [-0.228 ; 0.326] | Statistic: z = 0.35 | p-value: 0.7302 (ns)
- Effect: 2h | HAB - TR1
  - Magnitude: 0.034 [-0.303 ; 0.371] | Statistic: z = 0.23 | p-value: 0.8174 (ns)
- Effect: 2h | HAB - TR2
  - Magnitude: 0.101 [-0.224 ; 0.427] | Statistic: z = 0.73 | p-value: 0.8174 (ns)
- Effect: 2h | TR1 - TR2
  - Magnitude: 0.068 [-0.252 ; 0.387] | Statistic: z = 0.49 | p-value: 0.8174 (ns)

Correlation (Figure 4)

p-values are uncorrected for multiple testing.

- Effect: 0h_HAB | MKP3 - pERK
  - Sample Size: n = 10
  - Statistic: r = -0.042
  - p-value: 0.9090 (Significance: ns)
- Effect: 0h_TR1 | MKP3 - pERK
  - Sample Size: n = 10
  - Statistic: r = 0.689
  - p-value: 0.0270 (Significance: *)
- Effect: 1h_TR1 | MKP3 - pERK
  - Sample Size: n = 10
  - Statistic: r = 0.505
  - p-value: 0.1370 (Significance: ns)
- Effect: 1h_TR2 | MKP3 - pERK
  - Sample Size: n = 10
  - Statistic: r = -0.435
  - p-value: 0.2090 (Significance: ns)
- Effect: 2h_HAB | MKP3 - pERK
  - Sample Size: n = 8
  - Statistic: r = -0.435
  - p-value: 0.2810 (Significance: ns)
- Effect: 2h_TR1 | MKP3 - pERK
  - Sample Size: n = 8
  - Statistic: r = 0.116
  - p-value: 0.7840 (Significance: ns)
- Effect: 2h_TR2 | MKP3 - pERK
  - Sample Size: n = 8
  - Statistic: r = -0.773
  - p-value: 0.0250 (Significance: *)
- Effect: 0h_TR1 | TR1_exp - MKP3
  - Sample Size: n = 10
  - Statistic: r = -0.498
  - p-value: 0.1430 (Significance: ns)
- Effect: 1h_TR1 | TR1_exp - MKP3
  - Sample Size: n = 10
  - Statistic: r = -0.808
  - p-value: 0.0050 (Significance: **)
- Effect: 1h_TR2 | TR1_exp - MKP3
  - Sample Size: n = 10
  - Statistic: r = -0.371
  - p-value: 0.2910 (Significance: ns)
- Effect: 2h_TR1 | TR1_exp - MKP3
  - Sample Size: n = 8
  - Statistic: r = -0.243
  - p-value: 0.5630 (Significance: ns)
- Effect: 2h_TR2 | TR1_exp - MKP3
  - Sample Size: n = 8

- Statistic: r = -0.011
  - p-value: 0.9790 (Significance: ns)
- Effect: 1h_TR2 | TR1_exp - TR2_exp
  - Sample Size: n = 10
  - Statistic: r = 0.927
  - p-value: < 0.0001 (Significance: ***)
- Effect: 2h_TR2 | TR1_exp - TR2_exp
  - Sample Size: n = 8
  - Statistic: r = 0.873
  - p-value: 0.0050 (Significance: **)
- Effect: 0h_TR1 | TR1_exp - pERK
  - Sample Size: n = 10
  - Statistic: r = -0.406
  - p-value: 0.2450 (Significance: ns)
- Effect: 1h_TR1 | TR1_exp - pERK
  - Sample Size: n = 10
  - Statistic: r = -0.080
  - p-value: 0.8270 (Significance: ns)
- Effect: 1h_TR2 | TR1_exp - pERK
  - Sample Size: n = 10
  - Statistic: r = 0.272
  - p-value: 0.4470 (Significance: ns)
- Effect: 2h_TR1 | TR1_exp - pERK
  - Sample Size: n = 8
  - Statistic: r = 0.466
  - p-value: 0.2440 (Significance: ns)
- Effect: 2h_TR2 | TR1_exp - pERK
  - Sample Size: n = 8
  - Statistic: r = 0.358
  - p-value: 0.3840 (Significance: ns)
- Effect: 1h_TR2 | TR2_exp - MKP3
  - Sample Size: n = 10
  - Statistic: r = -0.594
  - p-value: 0.0700 (Significance: .)
- Effect: 2h_TR2 | TR2_exp - MKP3
  - Sample Size: n = 8
  - Statistic: r = 0.298
  - p-value: 0.4740 (Significance: ns)
- Effect: 1h_TR2 | TR2_exp - pERK
  - Sample Size: n = 10
  - Statistic: r = 0.405
  - p-value: 0.2460 (Significance: ns)
- Effect: 2h_TR2 | TR2_exp - pERK
  - Sample Size: n = 8
  - Statistic: r = 0.140
  - p-value: 0.7400 (Significance: ns)

Behavior analysis (Figure 5)

TeNOR (1st minute) - DI

Model Structure: DI ~ Group + Sex + (1|Cage)

Family: beta_family(link = 'logit')

DHARMa: Uniformity: p = 0.974 | Dispersion: p = 0.944 | Outliers: p = 1.000

- ANOVA:
  - Effect: Sex | Statistic: $\chi^2 = 1.19$, df = 1 | p-value: 0.2762 (ns)
  - Effect: Group | Statistic: $\chi^2 = 0.43$, df = 1 | p-value: 0.5138 (ns)
- Test vs Random: (0.5)
  - Effect: Latter_TR
  - Magnitude: 23.1% [9.7 ; 33.3]
  - Statistic: z = 3.23
  - p-value: 0.0025 (Significance: **)
- Test vs Random: (0.5)
  - Effect: Prior_TR
  - Magnitude: 17.5% [3.5 ; 28.9]
  - Statistic: z = 2.43
  - p-value: 0.0150 (Significance: *)

TeNOR (1st minute) - Total_exp_time

Model Structure: Total_exp_time ~ Group + Sex + (1|Cage)

Family: gaussian

DHARMa: Uniformity: p = 0.493 | Dispersion: p = 0.800 | Outliers: p = 1.000

- ANOVA:
  - Effect: Sex | Statistic: $\chi^2 = 1.27$, df = 1 | p-value: 0.2598 (ns)
  - Effect: Group | Statistic: $\chi^2 = 2.62$, df = 1 | p-value: 0.1053 (ns)
- Post-Hoc (Tukey):
  - Effect: Latter_TR vs Prior_TR
  - Magnitude: Diff(s) = 3.32 ± 2.05
  - Statistic: z = 1.62
  - p-value: 0.1053 (Significance: ns)

TeNOR (Full TS) - DI

Model Structure: DI ~ Group + Sex + (1|Cage)

Family: beta_family(link = 'logit')

DHARMa: Uniformity: p = 0.719 | Dispersion: p = 0.816 | Outliers: p = 1.000

- ANOVA:
  - Effect: Sex | Statistic: χ^2 = 0.18, df = 1 | p-value: 0.6746 (ns)
  - Effect: Group | Statistic: χ^2 = 4.98, df = 1 | p-value: 0.0257 (*)
- Test vs Random: (0.5)
  - Effect: Latter_TR
  - Magnitude: 14.2% [5.6 ; 22]
  - Statistic: z = 3.17
  - p-value: 0.0030 (Significance: **)
- Test vs Random: (0.5)
  - Effect: Prior_TR
  - Magnitude: 0.5% [-8.1 ; 9.1]
  - Statistic: z = 0.11
  - p-value: 0.9099 (Significance: ns)

TeNOR (Full TS) - Total_exp_time

Model Structure: Total_exp_time ~ Group + Sex + (1|Cage)

Family: gaussian

DHARMa: Uniformity: p = 0.773 | Dispersion: p = 0.800 | Outliers: p = 1.000

- ANOVA:
  - Effect: Sex | Statistic: χ^2 = 0.05, df = 1 | p-value: 0.8183 (ns)
  - Effect: Group | Statistic: χ^2 = 2.16, df = 1 | p-value: 0.1412 (ns)
- Post-Hoc (Tukey):
  - Effect: Latter_TR - Prior_TR
  - Magnitude: Diff(s)=7.74 ± 5.26
  - Statistic: z = 1.47
  - p-value: 0.1412 (Significance: ns)
